# A Treatise on Baths; Including Cold, Sea, Warm, Vapour, Gas, and Mud Baths; Also, of the Watery Regimen, Hydropathy, and Pulmonary Inhalation; with a Description of Bathing in Ancient and Modern Times

**Published:** 1850-06

**Authors:** 


Art. VIII.—*A Treatise on Baths; including Cold, Sea, Warm, Vapour, Gas, and Mud Baths; also, of the Watery Regimen, Hydropathy, and Pulmonary Inhalation; with a Description of Bathing in Ancient and Modern Times.* By John Bell, M. D., formerly Lecturer on the Institutes of Medicine, and on Materia Medica. Philadelphia: Barrington & Haswell. 1850.

There could scarcely be a more opportune publication than this. Between ignorance of the real and valuable uses of water, as a remedial agent, and the fear of being considered followers of Priessnitz, the profession has been avoiding the use of water, as a remedy, and in that way losing a valuable agent in curing many diseases. We have always regarded Currie's reports as one of the standard works, of practical medicine, that is destined to retain the confidence of all who read it. It is a book that deserves very careful study, and even the student who may read the excellent treatise before us, may turn from its classical, eloquent, and practical pages, to Currie's Reports, and feel his mind refreshed.

This work of Dr. John Bell, on Dietetical and Medical Hydrology, is the only treatise on the subject, in the English language, that may be considered a complete one. In addition to the valuable details of the work, that are the result of the author's own observations and reasonings, he has laid M. Gaudet's interesting Treatise on Sea Bathing, and the work of M. Rapou on Vapour Baths, under contribution. In this way, Dr. Bell says: "American readers are put in possession of a body of instructive matter not hitherto accessible in an English dress."

The former work of Dr. Bell: "On Baths and Mineral Waters," published in 1831, commanded the confidence of the profession throughout the country. The work on our table, however, is entitled to a much more extended approval. The author "has enlarged on all the topics of his former work, and has added new ones—so far as relates to baths and bathing." So great is the enlargement of the present work; it is so much more copious, thorough, and useful than the work of 1831, that it may almost, be regarded as a new treatise.

We are persuaded that there is too much neglect of the use of cold water, both in practical medicine, and in surgery. In many cases of fever, in various affections of the head and spine, and in a great variety of injuries that require surgery, cold water is invaluable. We have never been able to imagine how a physician can endure and encourage the agonies of a patient, in fever, whose parching thirst cries incessantly for cold water. There is scarcely any article of the Materia Medica more prompt and efficient, in relieving a febrile paroxysm; there is certainly no one more grateful to the patient. But an absurd prejudice, which has no foundation in fact, interdicts the use of cold water, when the patient is taking mercury. We have a great confidence in the powers of calomel, but if the foolish prejudice, of which we speak, had any authority over us, we should promptly give up the calomel, and depend upon the cold water as the greatest remedy of the two. How it ever happened that the notion came into use, that calomel salivates because cold water is used, we do not know. We have seen many more persons salivated, who confined themselves to warm drinks, than those who used the cold water. For twenty years we have urged upon patients the value of cold water drinks, while undergoing the use of calomel or blue mass, and so far from having seen any cause to regret it, we have found abundant sources of gratification. Any one who will use his reason, instead of depending on "old wives' fables," may find satisfactory reasons why the use of cold water drinks, in connection with mercurial treatment, in febrile or inflammatory conditions, will prevent salivation. The whole subject may be thrown into a syllogism, consisting of well established truths—if calomel purges freely, it is not very likely that it will act on the salivary glands—it cannot purge well, if the febrile or inflammatory symptoms are above the secreting point, and the lancet itself is not a more powerful agent, for reducing the excessive actions of the arterial system, than cold water—the sequence is clear, cold water can and does prevent salivation, but ptyalism may come on, despite of the cold drinks; we know it often does, when they are avoided.

We are much gratified with the remarks of Dr. Bell on the use and various values of cold water drinks, and we hope his sentiments will exert a wide-spread influence over the profession. A work of this kind was much needed on this very point, and it could not have been undertaken by a more competent man. We are wedded to the use of cold drinks, by personal experience. For thirty years and upwards, we have undergone an amount of toil, endurance, and exposure that few have experienced. We have thrived on four hours sleep in the twenty-four; we never knew a leisure hour yet, we have arduously engaged for twenty years in the practice of medicine, in drenching rains, snow storms, sultry heats, repeated losses even of the pittance of sleep claimed as due, and through all, have maintained almost uninterrupted health. And we know no more of the taste of alcoholic drink, than if there never had been such a thing known. Not a drop of anything of the kind has ever been in our mouth. Nor, save at the communion table, do we know the taste of wine. But any deficiences of these privileges have been more than made up in an intense love of cold water, and no toper ever revelled in the love of his intoxicating dram, more than we do in the love of a glass of cold water. To the free use of that drink, we owe unusual health, in the midst of the many physiological sins, we are forced to commit.

We love cold water in the same way that old Peale said he loved brandy—internally, externally, and eternally.

We cordially commend the work of Dr. John Bell on "Dietetical and Medical Hydrology," to the profession. It is a work of rare and unusual merit. It is classical in its style, pleasing and instructive in its varied details, and exceedingly practical in all its departments.

We owe many thanks to the publishers, Barrington & Haswell, for the very excellent manner in which they have published this work. It is one of the most beautiful publications we have seen; excelling in typography and tastefulness, even Putnam's editions of Washington Irving's works. This beautiful volume is as well fitted for display in a lady's *boudoir*, as upon the shelves of a library, and the mind drinks in the pleasant knowledge of the author, with additional delight arising from the tastefulness of the goblet from which it flows.

These works, Maclise's Surgical Anatomy, and Bell on Baths and Watery Regimen, may be found in the excellent collection of medical books, at the bookstore of Beckwith & Morton.

